# What can Designing Learning-by-Concordance Clinical Reasoning Cases Teach Us about Instruction in the Health Sciences?

**DOI:** 10.5334/pme.898

**Published:** 2023-05-18

**Authors:** Nicolas Fernandez, Marie-France Deschênes, Haifa Akremi, Lise Lecours, Vincent Jobin, Bernard Charlin

**Affiliations:** 1 Department of Family Medicine and Emergency Medicine, Faculty of Medicine, Université de Montréal, CA; 2 Faculty of Nursing, Université de Montréal, CA; 3 Continuous Professional Development, Faculty of Medicine, Université de Montréal, CA; 4 Le-Cours, Inc. President and e-learning specialist, CA; 5 Department of Surgery, Faculty of Medicine, Université de Montréal, CA

## Abstract

**Introduction:**

Learning-by-concordance (LbC) is an online learning strategy to practice reasoning skills in clinical situations. Writing LbC clinical cases, comprising an initial hypothesis and supplementary data, differs from typical instructional design. We sought to gain a deeper understanding from experienced LbC designers to better support clinician educators’ broader uptake of LbC.

**Methods:**

A dialogic action research approach was selected because it yields triangulated data from a heterogeneous group. We conducted three 90-minute dialogue-group sessions with eight clinical educators. Discussions focused on the challenges and pitfalls of each LbC design stage described in the literature. Recordings were transcribed and analyzed thematically.

**Results:**

We identified three themes by thematic analysis about the challenges inherent in designing LbC that are unique for this type of learning strategy: 1) the distinction between pedagogical intent and learning outcome; 2) the contextual cues used to challenge students and advance their learning and 3) the integration of experiential with formalized knowledge for cognitive apprenticeship.

**Discussion:**

A clinical situation can be experienced and conceptualized in many ways, and multiple responses are appropriate. LbC designers use contextual cues from their experience and combine them with formalized knowledge and protocols to write effective LbC clinical reasoning cases. LbC focuses learners’ attention on decision-making in grey areas that characterize the nature of professional clinical work. This in-depth study on LbC design, indicating the integration of experiential knowledge, might call for new thinking about instructional design.

## Introduction

The need to make clinical decisions based on available information is at the core of modern professional practice in Health Care [1]. Many strategies have been described in the literature for teaching how to make clinical decisions. Overall, strategies are based on self-explanation [2] requiring students to reflect and explain while engaging with clinical cases to deepen their understanding and monitor knowledge and skill acquisition. One approach uses concept mapping [3,4] to support students’ knowledge structuring by comparing and contrasting typical clinical features of illnesses. Another is based on deliberate reflection, an approach that encourages and guides reflection during the diagnosis of clinical cases, requiring students to compare alternative diagnoses for a given case systematically [5]. The purpose is to foster the development of mental representations of the bio-medical knowledge related to clinical cases, making it easier to arrive at the appropriate diagnosis in the future.

**Figure 1:**
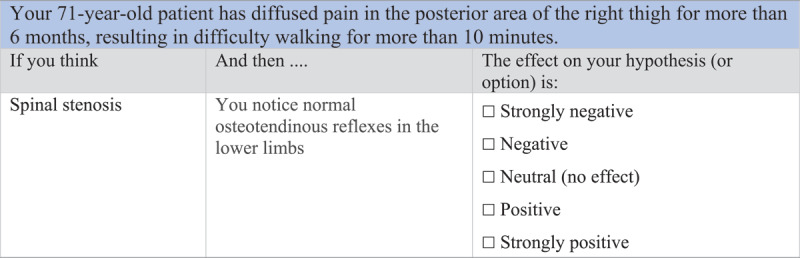
Clinical reasoning case from physiotherapy using a 5-point Likert scale. Adapted from Charlin, Deschênes & Fernandez, 2021.

Learning-by-concordance (LbC) [6,7] is an online learning strategy that makes learners practice reasoning skills in contexts of uncertainty. A typical LbC learning task presents a clinical case and an initial hypothesis about it in brief terms. In this way, the instructional designer who chooses LbC seeks to focus the learners’ attention on a specific line of reasoning. Subsequently, a new piece of information – supplementary data – is presented to trigger a reflective process that challenges the initial hypothesis. Figure 1 presents a typical LbC clinical reasoning case [7].

The cognitive dissonance generated by the supplementary data prompts the learner to revisit the suggested initial hypothesis and engage reflexively. Thus, LbC clinical reasoning cases are designed to deliberately trigger system 2 thinking, as described by dual-process theory [8].

LbC has been increasingly used in Health Sciences education in three distinct formats, each focusing on clinical reasoning [9], visual perception [10,11], or attitudes and behaviors (professional judgment and clinical ethics) [12]. LbC has been used in undergraduate medical training, post-graduate training [13], and continuous medical education [10] (see Fernandez et al. [6] for further details). Significant knowledge gaps exist regarding the LbC instructional design process – namely, how to write the clinical reasoning case to trigger appropriate reflexive (system 2) learning. We were interested in studying the LbC design process to help instructional designers develop compelling LbC clinical reasoning cases. Specifically, we sought to gain a deeper understanding of how LbC reasoning cases are written (e.g., the initial hypothesis and the supplementary data) by clinician educators who have experience designing LbC clinical cases in the recent past.

## Methods

### Setting and Participants

The study took place at a research-based university in North America. Having been involved in multiple instructional design projects with clinician educators learning to use LbC, we had ready access to participants who had designed LbC training tools during the last five years (2016–2021). Hence, it was natural to recruit these clinician educators by network sampling to understand better how LbC reasoning cases are written. The inclusion criteria were that all participants share meaningful and recent experiences designing LbC. In total, 10 people agreed to participate, including eight educators in medicine, physiotherapy, and nursing and two researchers (see Table 1).

**Table 1:** Study Participants.


	PARTICIPANTS (PARTNERS AND RESEARCHERS)	PROFESSION	EDUCATIONAL CREDENTIALS	EXPERIENCE IN LBC

1	Male	Surgery	Ph.D. in Education	Inventor of the Script Concordance Test and Learning by Concordance

2	Female	Nursing	Ph.D. in Nursing Education	Thesis work on LbC in nursing developed LbC tools for the nursing profession

3	Male	Physiotherapy	Ph.D. in Education	Developed SCT for the Physiotherapy program

4	Female	Surgery	M.A. Education	Developed learning by concordance of judgment tool for clerkship

5	Female	Speech therapy	Orth. (SLP)	Developed an interprofessional tool for Dysphagia

6	Female	Pediatrics	Ph.D. in Clinical Ethics	Developed an LbC for Clinical Ethics

7	Male	Internal medicine	Clinician educator and Director of CME	Directed the development of LbC in CME tools for multiple professions

8	Female	Nutritionist	M.A. in Education	Online producer of LbC for continuous education in Health Care

9	Female	Physiotherapy	Ph.D. student in Rehabilitation science	Provided clinician insight for study and facilitated the discussions

10	Male	Higher Education	Ph.D. in Educational sciences	Lead researcher of the project and author of multiple papers on LbC


### Study Design

A dialogic action research design [14] was selected because it yields enriched and triangulated data from a heterogeneous group. The dialogue group technique stems from practical focus groups [14,15] modified to optimize exchanges among participants who share a common experience. Part of an interpretive qualitative research approach [16], the dialogue group engages in on-the-spot reflection by individuals followed by facilitated discussions to gain a deeper understanding [14]. These discussions mitigate potential bias from self-reported recollections because others confront each individual’s utterances within the group. The facilitator (research team member) asked questions to focus participants’ attention on their thoughts and guided the ensuing dialogue to unpack ideas as thoroughly as possible. At the end of each session, the facilitator summarized ideas, and participants could modify them if needed and signify their agreement. This procedure ensured the validity and credibility of the reported data [16,17].

### Data Collection and Analysis

Three dialogue group sessions were conducted with the same participants via Zoom video conference (Zoom Video Communications, San Jose, CA) on the 24, 25 February, and 10 March 2021. Because the group dialogue requires ample time, we initially planned for four 90-minute sessions so that issues could be explored fully. However, after the third 90-minute session, we considered having exhausted our questions and collected enough data to proceed to the analysis phase. The interview guide (see appendix) was based on the main stages of LbC problem design described in Charlin et al. AMEE guide [7].

During the discussion sessions, participants were asked to remember their thoughts while working on a specific LbC development stage. An example of an LbC reasoning case was provided to guide discussions and keep participants focused on the given stage. Specifically, participants were asked to reconstruct their thinking processes as they wrote the clinical case components. The dialogue group technique allows participants to listen, share and compare their experiences, thereby contributing to a broader understanding of the process. In this way, a nuanced and agreed insight about the pedagogical challenges at each design stage emerges.

The dialogue group exchanges were recorded and transcribed. Thematic analysis [17,18] on the verbatim sections was conducted independently by three authors (MFD, BC, and NF) using the following steps: 1) familiarization with the study data; 2) generation of initial codes; 3) identification of themes from aggregate codes; 4) revision of themes; 5) definition and description of themes; and 6) production of the report [18]. The researchers (MFD, BC, NF, and AK) held two meetings between the 2^nd^ and 3^rd^ sessions to compare coding and discuss discrepancies. These initial findings were shared with the participants during the 3^rd^ session, who checked and validated them. The final report was shared with participants for comments; all signified their agreement with our conclusions, and none had anything to add.

### Reflexivity

We are conscious of the risk of “groupthink” [19] bias – all ten participants have worked closely for many years on LbC tools. However, because LbC is an emergent practice in medical education, there are no colleagues outside our institution with equivalent mastery that we could turn to for external validation. We mitigated this by explicitly asking participants to focus on how they completed the well-identified instructional design stages. Finally, the facilitator deliberately guided group discussions to avoid appraising LbC’s advantages or possible benefits.

### Funding and Ethical Considerations

The study received funding from the Social Sciences and Humanities Research Council of Canada Partnership Engagement Grant in 2020. The study protocol received approval from the University’s Science and Health Research Ethics Committee in October 2020 (#20-118-D).

## Results

We identified three themes from the data: 1) the importance of pedagogical intent for LbC design, 2) the contextual cues used to challenge students to advance their learning, and 3) The integration of experience and formalized knowledge. The following section presents each theme and supporting quotes from the verbatim data.

### The necessary distinction between pedagogical intent and learning outcome

Pedagogical intent, as opposed to learning outcomes, emerged as central to designing LbC, akin to instructional guidance used by Merrill [20]. The intent is the underlying purpose of designing a given clinical case expressed by participants as what they want the “learner to think about” (668–669, Session 2, P1). The underlying instructional design challenge for participants was to identify clinical cases that would make students reflect: these are cases with “grey areas [where there is] uncertainty.” (345–346, Session 1, P2) Participants agreed that cases, where the outcome is hard to predict, are the best for Learning-by-concordance.

Participants agreed that LbC allows them to provide learning opportunities where there is no right or wrong answer or awkward and uncomfortable situations where values conflict. The overall purpose is that when these situations occur in real life, “students already have some idea how to manage them in accordance to their values, all the while maintaining a professional stance” (338–341, Session 1, P4).

Once LbC designers identify a clinical case, they write a brief description and an initial hypothesis. The brief description allows them to focus on the reasoning they want their students to enact (see quote 1). Participants spoke of the initial hypothesis and how it should flow naturally from the clinical case to guide learners’ thinking and prime them for the supplementary data to engage in deep reflection.

### The contextual cues used to challenge students to advance their learning

All participants reported difficulty selecting the supplementary data to fit the initial hypothesis. There are too many possible pieces of information to choose from – a clinical situation can be experienced and conceptualized in multiple ways (see quote 2). Participants shared with us that their choices had been arbitrary because there was no formal guidance on this aspect of LbC design.

As framed in the dialogue group, the issue is that the same clinical situation can be experienced differently. Quotes 2 and 3 in Table 2 illustrate how participants realized there could be multiple ways to understand a problem and how contextual cues can change a clinical situation entirely. Even in seemingly straightforward cases, there are still areas for “adjustment between what I know and what is appropriate.” (129–130, Session 1, P5) This participant spoke of “personal preferences” about clinical practice. Participants suggested that within any clinical situation, between its “messiness and unpredictability” and the general principles and reflective skills required, it is the “instructor’s job to make this dichotomy digestible” (407–415, Session 1, P3) for their students (see quote 4).

**Table 2:** Dialogue group quotes in support of themes identified by researchers.


QUOTE	VERBATIM	INTERPRETATION

1	The hypothesis helps to reduce the clinical situation to the core, even though there might be plenty of other aspects [that could be tackled]. One of us said that sometimes we feel that complexity can be minimized…but at some point, you must delve a little deeper into the clinical situation. Otherwise, you are tempted to talk about many topics and have to choose only one. I think the initial hypothesis [helps us] strangle the vignette a little bit to fit in with our intent. (489–494, Session 2, P6)	Pedagogical intent is central to LbC as it allows designers to identify what cognitive processes they would like students to work on. A critical step is the choice of initial hypothesis.

2	We found many situations with no single answer and many ways to understand the problem. So [we] tried to expose our students to a variety of possibilities or a variety of responses and force them to confront how they would respond or position themselves in the context of that situation. (5–9, Session 1, P6)	A clinical situation can be experienced and conceptualized in multiple ways.

3	[depending on the context, you] react differently. For instance, if the clinical situation is that of a clerk entering the operating theater and notices that the surgeon is drunk: what the clerk should do is report it, obviously. From one point of view, it is the normal thing to do. But, if the clerk wants to be admitted to surgery [residency] and he is working with that attending [surgeon] who will grade the rotation…the clinical situation is not the same anymore. (344–351, Session 1, P6)	Contextual cues provide nuance to a clinical case. LbC designers use this nuance to challenge students to advance learning

4	I think the richness comes from the experience within a specific context. I think the instructional designer has to make the material digestible while attempting to present the diversity of possibilities for the learner. So, I think LbC design, when there is such diversity, makes it clear that diversity comes from the context. In this specific instance, it is appropriate to proceed like this, but in another, it is not. (407–415, Session 1, P3)	Instructional design in LbC aims to highlight the diversity of responses and strengthen appropriate reflexive skills

5	Regarding our experience in dysphagia, [we realized] that what is taught at the university is one thing, and what happens in clinical settings [is another]. (238–241, Session 1, P5)	The gap between formalized knowledge and practice can be embedded in instructional design

6	I think one of the reasons why we selected LbC is because it is a form of cognitive apprenticeship. Learners greatly appreciate the opportunity to calibrate their thinking on that of experts, seeing how experts respond in a given situation. (91–95, Session 1, P3)	By revealing their clinical reasoning, instructors support the development of this skill in students.


For participants, the core instructional design issue is to balance specific clinical situations and the general features of clinical reasoning. The supplementary data they choose forces them to narrow the focus: “We can see better what we want our learners to think about” (668–669, Session 2, P1) and is perceived as an opportunity to challenge students to develop their thinking and position themselves in a specific situation.

### The integration of experience and formalized knowledge

Participants explicitly linked designing Learning-by-concordance cases and knowledge acquired through experience. At the outset, they framed the problem in the following dichotomy: what they teach at the university and how they do things in hospitals (see quote 5).

Given this, and their role as educators, participants pointed to the need to integrate knowledge derived from their clinical experience with general concepts or formalized knowledge that learners should acquire.

The challenge of integrating types of knowledge is mitigated when LbC cases are designed in groups, which is often the case. Participants said that working with colleagues enriched the contextual aspects of the LbC case and provided more effective learning for the students.

Finally, participants shared with us that students appreciated the LbC tool because it showed them how clinicians or expert clinicians think and resolve problems (see quote 6). The cognitive apprenticeship, as alluded to by this participant, reflects the idea that much of the content material embedded in LbC is the knowledge that arises from the application of formalized knowledge and the necessary adjustments in practice.

## Discussion

Our results indicate that LbC provides an opportunity for the instructor to support the development of learners’ reasoning skills by 1) identifying their pedagogical intent as distinct from learning outcomes, 2) introducing contextual cues to challenge reasoning, and 3) integrating knowledge gained from experience with the formalized concepts and practice guidelines learners need to master. From an instructional design perspective, the instructor’s intent provides overall guidance, the initial hypothesis triggers a reflective process, and the supplementary data provides the necessary contextualization to challenge the learner beyond the straightforward response.

While shining light into the nature of LbC, our results offer an empirical glimpse into step-by-step guidance, as described by Merrill [20], through acquiring and applying new knowledge. LbC reasoning cases allow instructors to blend contextual and general knowledge, facilitating integration into learners’ long-term memory [7,21]. Also, LbC focuses on applying knowledge in response to complex and uncertain situations. To achieve this from an instructional standpoint, clinician educators must refer to their clinical experience, i.e., the specialized and highly contextualized knowledge [22] they have accumulated to write compelling reasoning cases.

The distinction between learning outcome and pedagogical intent surprised us, given the greater attention devoted to student learning outcomes by Health Sciences education researchers. Our study allowed us to refocus on the importance of pedagogical intent in the instructional design process. Intent, according to results, includes broadly defined outcomes (i.e., to reflect on conflicting values or to exercise critical judgment when interpreting a negative *d-dimer* reading in pulmonary embolism). There is less emphasis on defining precise and measurable learning outcomes useful for assessment purposes. Instead, the goal is to support reasoning in situations where multiple conceptualizations and courses of action are possible.

LbC cases, particularly Concordance-of-judgment [6] cases, are distinct from such tools as the Situational Judgment Test [23] because different but appropriate responses are possible. Our study results provide further insight into this distinctive aspect. Participants debated the difficulty and importance of supplementary data to reframe the initial hypothesis, which generally reflects established practice and protocols. Supplementary data can challenge a given initial hypothesis, each leading to different outcomes, mimicking clinical practice’s complex and uncertain nature. Participants insisted that what mattered was for students to enact nuanced and adaptive clinical reasoning.

While the reasoning case may be generic (diagnose, etc.), its application in a specific context can be tailored to advance student learning, facilitating retention and transfer to similar settings [24]. Furthermore, because LbC calls for instructors to share their experiential knowledge (e.g., a validation phase), students are ultimately exposed to multiple rules of thumb or tricks of the trade from experienced clinicians when building their future practice.

LbC reasoning cases present theory and practice simultaneously rather than sequentially, as in traditional instructional design approaches such as the Dick and Carey model or 4C/ID. Embedding a specific context in the supplementary data, picked from instructors’ experience, affords the LbC instructor turned designer a nuanced impact on student learning. Consequently, the inherent value of LbC lies in that learners apply evidence-based and experience-based knowledge with guidance from seasoned clinicians accustomed to making decisions in grey areas of clinical practice.

### Study limits

The limits of our study concern the data collection technique, which was carried out via Zoom. Large parts of the body language were thus not available to us. However, familiarity and ease were quickly established over the three sessions because the study team and partners had worked together before. We feel that participants were sufficiently sincere with us in divulging the challenges and pitfalls they had experienced with LbC design. The selected data collection technique had the appropriate safeguards against self-reported bias. Thus, we are confident that our data had acceptable levels of validity and credibility.

Furthermore, this is a limited group of clinicians, and their experiences with LbC were influenced by the researchers involved in the project. Hence, the possibility of “groupthink” is real. LbC is not widespread, and the variety of ways it is used have not been documented, which provides some justification for the modest size sample. Hence, we consider our study an exploratory venture, seeking to describe rather than appraise.

## Conclusion

The challenge for LbC designers is to create compelling clinical reasoning cases to help students develop appropriate responses in clinical situations that are complex and uncertain. LbC is best suited to practice reasoning in clinical cases where more than one response is appropriate. This feature poses unique challenges for LbC design because the focus is on the reasoning process rather than the correct answer.

Far from suggesting that Learning-by-concordance is the silver bullet, our study contributes to our understanding of instructional design in Health Sciences education. LbC design teaches us that besides conveying knowledge, instructors also guide learners through grey areas of clinical practice by modeling their processes acquired through experiential learning.

## Appendix

### Group dialogue interview guide

#### Session 1

How can we document the needs of the community of practice?

- Who needs this learning? (students, residents, practicing professionals…)
- What are the learning needs of this group?
- How can you determine their needs?
- What means do you use to document the issues of the community of practice?
- What are the critical elements/issues to determine in the field?
- What are your benchmarks for determining issues in the field?
- What are the critical elements of reflection in the domain?
- How can you identify the point(s) on which one would like to establish reflection/reasoning among health professionals?
- What means do you use to document the points of reflection?
- How do you identify these key elements?

#### Session 2

What do you want to know about the process in question?

Present an example to illustrate: e.g., present one or more large situations and the vignettes that were produced.

Clinical Practice Theme:

During a pandemic, masks are mandated to be worn in hospitals.

Clinical situation: In the hospital setting, for the past week, it has been required that healthcare professionals wear masks when in contact with patients and remove them in common areas such as the cafeteria.

- What are the issues/thoughts around wearing masks?
- What is the experiential knowledge that allows you to move from a broad situation to an FpC vignette

Expertise sharing:

- Developing a situation that is neither too long nor too short: how do you find the right balance to include information relevant to the instructional intent of the situation?
- Tell us about your method for summarizing/describing the clinical situation in 2–3 sentences
- How do you ensure that the situation is learning and reflective?
- How do you envision this process?
- Are there any markers for this process?

Synthesis and validation of data

- As I understand it, the process involves A, B, and C…
- Adjust synthesis based on feedback
- What did you find challenging to address/facilitate in the discussions?
- Set the next workshop date
- Close the workshop

#### Session 3

What do we want to know about the process in question? Generating additional data that solicits reasoning. Present an example to illustrate: e.g., present one or more vignettes generated from clinical situations.

Vignette: In the hospital setting, for the past week, healthcare professionals have been asked to wear masks when in contact with patients and to remove masks in common areas such as the cafeteria.

If you were thinking of removing your mask in common areas

And you find that in common areas, people do not respect the 2 m distance

Expertise Sharing – Hypothesis:

- Describe the process of developing a hypothesis
- On what basis (what criteria) do you determine the hypothesis in a clinical situation? Explain

Expertise sharing – supplementary data:

- Tell us about your reasoning process for determining the supplementary.
- What data are essential in thinking about this topic?
- How do you select data to prompt participants’ thinking?
- How do you pair hypothesis and data to guide thinking?

Sharing expertise: linking the hypothesis to additional data to create questions:

- What strategy do you take to get participants to generate all the possibilities of answer choices? Explain

Expertise sharing: Generating the different possibilities of the Likert scale to answer the questions:

- How do you ensure you cover all the response choices on the Likert scale?
